# HAIC versus TACE for patients with unresectable hepatocellular carcinoma: A systematic review and meta-analysis

**DOI:** 10.1097/MD.0000000000032390

**Published:** 2022-12-23

**Authors:** Junguo Liu, Jinjuan Zhang, Yijun Wang, Guiming Shu, Cheng Lou, Zhi Du

**Affiliations:** a The Third Central Hospital of Tianjin (The Third Central Clinical College of Tianjin Medical University), Tianjin Institute of Hepatobiliary Disease, Tianjin Key Laboratory of Extracorporeal Life Support for Critical Diseases, Artificial Cell Engineering Technology Research Center, Tianjin, China.

**Keywords:** Hepatic arterial infusion chemotherapy, transarterial chemoembolization, unresectable hepatocellular carcinoma

## Abstract

Hepatic arterial infusion chemotherapy (HAIC) and Transarterial chemoembolization (TACE) both showed good local efficacy in advanced or unresectable hepatocellular carcinoma (HCC). We performed a systematic review and meta-analysis to compare the effect of HAIC with TACE in patients with unresectable HCC.

**Methods:**

Clinical trials, which were about HAIC or TACE in Patients with unresectable HCC, were identified by searching PubMed, Medline, and EMBASE from January 2010 to March 2022. A meta-analysis was performed to analyze HAIC in comparison with TACE. Treatment response, 1-year overall survival (OS), 2-year OS and serious adverse events were evaluated in this meta-analysis.

**Results:**

This meta-analysis included 6 studies. Objective response rate or Partial response in the HAIC group was significantly more than that in the TACE group (*P* < .05). But, stable disease showed no difference between the 2 groups (*P* = .52). Disease control rate in the HAIC group was better than that in the TACE group (*P* < .05). Progressive disease in the HAIC group was less than that in the TACE group (*P* < .05). In 1-year OS, there was no significant deterioration between the 2 groups (*P* = .53). There was not significant difference in 2-year OS between the 2 groups (*P* = .05). serious adverse events in the HAIC group was significantly less than that in the TACE group (*P* < .05).

**Conclusion:**

To some degree, HAIC may be a better therapeutic method in patients with unresectable HCC than TACE.

## 1. Introduction

Hepatocellular carcinoma (HCC) was ranked fourth by number of incident cases and the third by number of cancer-related deaths worldwide.^[1,2]^ Unfortunately, most patients with HCC are in advanced or unresectable stage. ^[3,4]^ For patients with large or huge HCC, who are not suitable for surgical resection, the treatment remains a major challenge.^[5]^ In addition, large or giant HCC was usually unresectable due to insufficient surgical margins, a residual liver volume estimated less than 30% after resection, or large vessel invasion.^[6]^ Transarterial chemoembolization (TACE) and hepatic arterial infusion chemotherapy (HAIC) showed good local efficacy in advanced or unresectable HCC.^[7]^ However, some studies about HCC have shown that TACE is effective treatment for patients with portal vein tumor thrombus (PVTT) alone and TACE can improve the 1-year survival rate.^[8,9]^ HAIC, which is different from TACE, can provide stable and continuous local chemotherapy drugs^[10]^ and has less toxicity to surrounding liver issue.^[11]^ HAIC is also beneficial for HCC with Vp3-4 PVTT.^[12]^ For the advanced HCC, HAIC is not recommended in the guidelines of the American Association for the Study of Liver Diseases, the National Comprehensive Cancer Network, the European Society of Liver Diseases and the Asia Pacific Association for the Study of Liver Cancer.^[13–17]^

In this article, we compared the efficacy of HAIC with TACE in the treatment of unresectable HCC, and systematic reviews and meta-analysis were carried out. Using evidence-based medicine, a meta-analysis including 6 clinical literatures was conducted to provide a more reasonable clinical basis for the clinical treatment options.

## 2. Materials and Methods

This study is a systematic review and Meta-analysis, which does not require a statement indicating that the study was approved by the Institutional Review Board nor comparable formal research ethics review committee by providing the decision/protocol number of the approval. However, we have acquired a PROSPERO (Registered)ID, which is CRD42022313819.

### 2.1. Literature retrieval strategy

Medline, EMBASE, and PubMed electronic databases were searched for literatures from January 2010 to March 2022. The following keywords, such as “Transarterial chemoembolization’’, “TACE,” “Hepatic Arterial Infusion chemotherapy,” “HAIC,” “unresectable,” “HCC”, and “hepatocellular carcinoma,” were used. In addition, all relevant publications, review articles and lists of citations included in the study, were manually searched. The language was restricted to English.

When 2 reports overlapped, only more detailed report was enrolled. We contacted the authors to obtain more details of the cases which they reported, appropriately.

### 2.2. Data extraction and quality assessment

The authors (Junguo Liu, Jinjuan Zhang, and Yijun Wang) reviewed and screened the enrolled articles. The data, such as the number of patients, treatment response and Survival and Safety, were extracted. Newcastle–Ottawa Scale was used to assess the quality of nonrandomised studies. And Jadad rating scale was used to evaluate the quality of randomized controlled clinical trial.

### 2.3. Study inclusion and exclusion criteria

The selected articles had to meet the following criteria:

1. All selected patients with unresectable HCC; 2. Studies about cases of treatment response (complete response; partial response (PR); stable disease (SD); disease control rate (DCR); objective response rate, objective response rate (ORR); progressive disease (PD), cases of Survival progression-free survival; overall survival (OS) and cases of Safety (adverse events (AEs); serious adverse events (SAEs)). 3. Randomized or nonrandomized controlled studies conducted or published for many years. 4. Clearly define the sample size, such as the number of cases in HAIC group and TACE group. 5. Based on HAIC and TACE techniques. 6. Only English literature was enrolled in this study. 7. Common AEs, such as pain, vomiting, fever, nausea, happen usually in the HAIC group or TACE group and can be controlled by medical treatment, but not the same as SAEs including death, progressive deterioration of liver function, or liver failure.

Exclusion criteria were as follows:

1. letters to the editor; 2. study protocols; 3. conference abstracts; 4. case reports; 5. animal studies; 6. editorials; 7. posters.

### 2.4. Statistical analysis

The dichotomous data was assessed based on odds ratio (OR) with 95% confidence intervals (CIs). All analyses was performed using the Review Manager 5.3 software. Study-to-study variation was assessed by suing the chi-squared statistic. A fixed-effect model was used when no heterogeneity. And whereas in the presence of significant heterogeneity, a random-effect model was performed. The funnel plot and Begg’s test for asymmetry were applied to assess the possibility of publication bias among the studies. Statistical significance was set at a *P* level of .05.

## 3. Results

### 3.1. Study population

**Figure 1.**
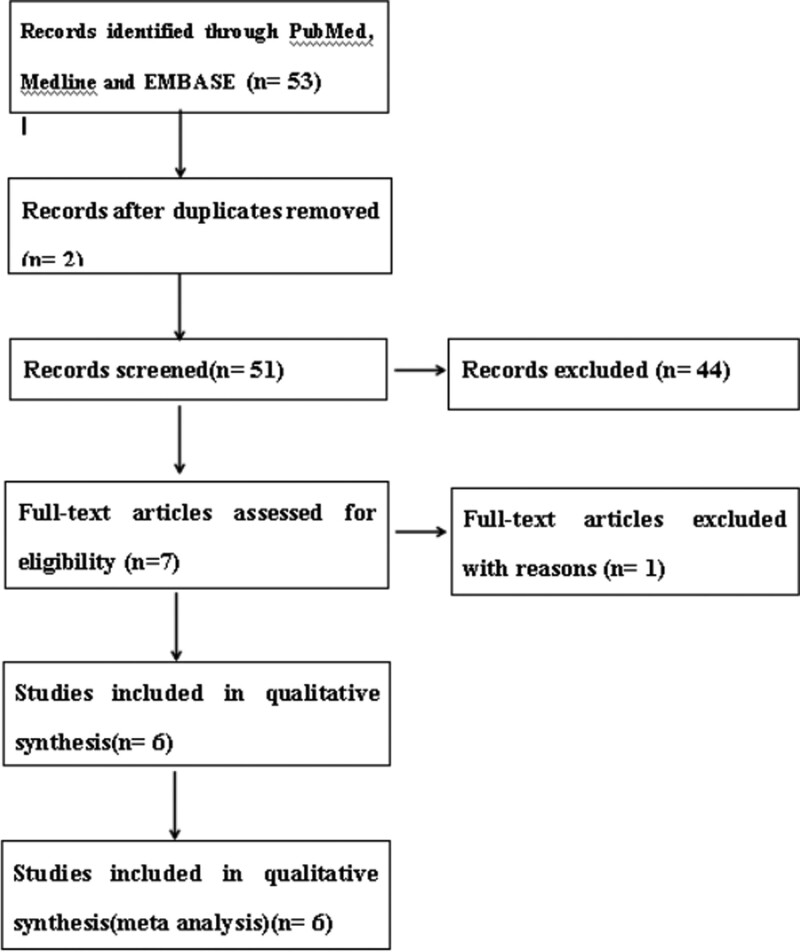
Diagram of the search strategy.

In the first search, 53 studies appeared and then 2 duplicates were removed. According to the study inclusion and exclusion criteria, 44 studies were excluded. After assessing full-text articles for eligibility, only 6 studies ^[18–23]^ were included in the meta-analysis (Fig.1). A total of 558 patients with unresectable HCC underwent HAIC (n = 281) or TACE (n = 277) treatment from January 2010 to March 2022. These studies including one randomized controlled trial and 5 nonrandomized controlled trials were all characterized as high quality (Table 1). Objective response rate (ORR) includes complete response and PR. There were not any complete response in the HAIC group or TACE group. So, ORR is the same as PR.

**Table 1:** Methodological quality of studies included in meta-analysis.

Author	Year	Treatment(cases)		Study design	Quality evaluation Score*
		HAIC	TACE		
Chao An	2021	92	68	Nonrandomized controlled trials	5*
Hee Yeon Kim	2010	36	31	Nonrandomized controlled trials	6*
Jungang Hu	2020	22	24	Nonrandomized controlled trials	6*
Min-Ke He	2017	38	41	Nonrandomized controlled trials	6*
Qi-jiong Li	2021	159	156	Randomized controlled trials	3†
Wei-Lun Tsai	2020	38	41	Nonrandomized controlled trials	5*

* According to the Newcastle–Ottawa Scale for assessing the quality of nonrandomised studies, >=5* is divided into meta-analyses.

† According to the quality of randomized controlled clinical trial evaluation criteria (Jadad rating scale) score> = 3 is divided into high-quality research.

### 3.2. Meta-analysis

Regarding treatment response (ORR, PR, SD, DCR, PD), 1-year OS, 2-year OS and SAEs, HAIC was compared with TACE in patients with unresectable HCC by meta-analysis.

### 3.3. Treatment response

#### 1.3.3. Objective response rate (ORR) or PR.

**Figure 2.**
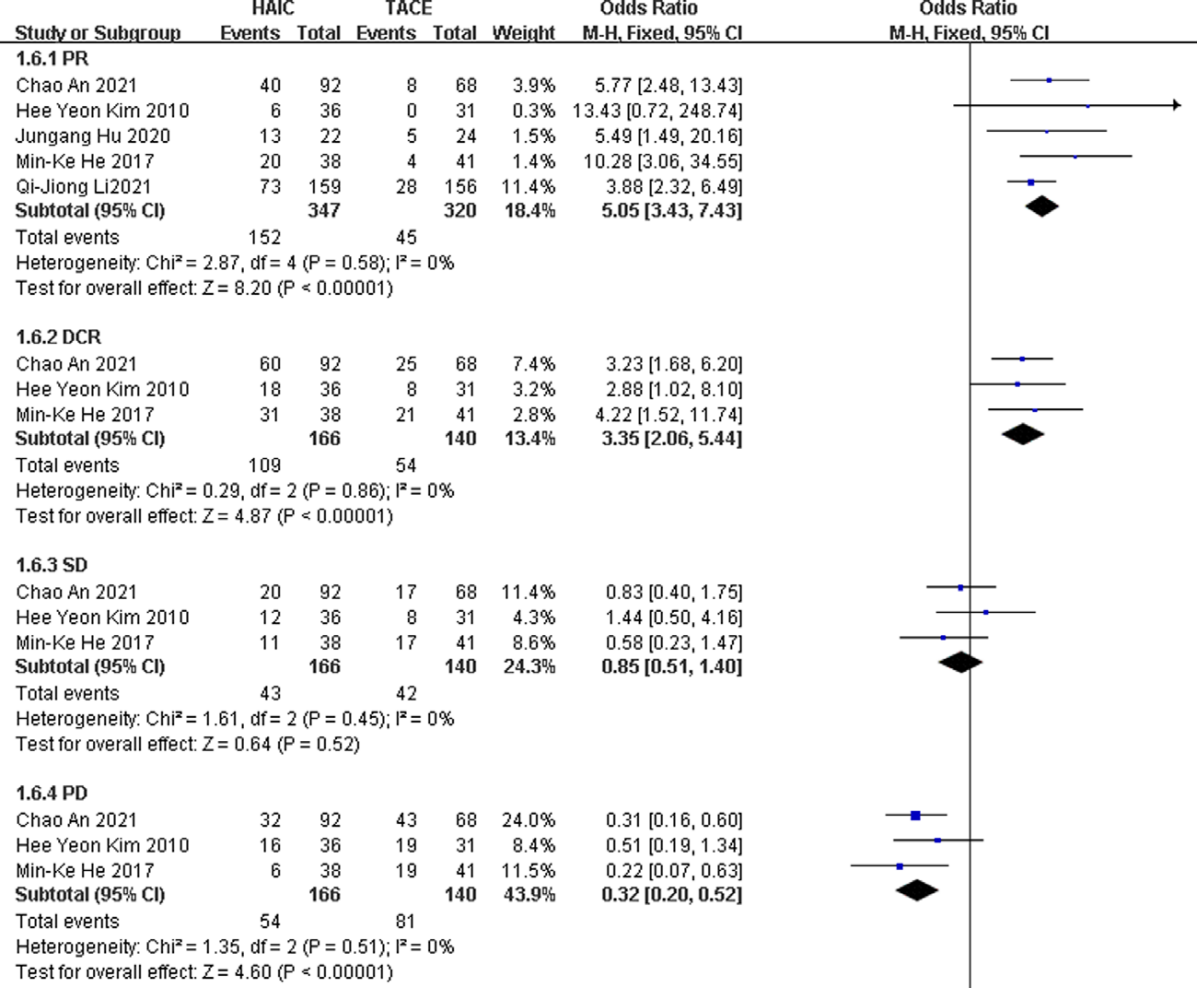
Fixed-effects statistical model of odds ratio (OR) for Treatment response after HAIC versus TACE.

The chi-squared test of heterogeneity was not significant from 5 studies (*P* = .58). Fixed-effect statistical model was performed. ORR or PR in the HAIC group was more than that in the TACE group with a combined OR of 5.05 (95% CI, 3.43, 7.43; *P* < .05) (Fig. 2).

#### 2.3.3. SD.

The chi-squared test of heterogeneity was not significant from 3 studies (*P* = .45). Fixed-effect statistical model was adopted. There was not significant difference in SD between the 2 groups with a combined OR of 0.85(95% CI, 0.51, 1.40; *P* = .52)

#### 3.3.3. DCR.

The chi-squared test of heterogeneity was not significant from 3 studies (*P* = .86). Fixed-effect statistical model was adopted. DCR in the HAIC group was better than that in the TACE group with a combined OR of 3.35 (95% CI, 2.06, 5.44; *P* < .05).

#### 4.3.3. PD.

The chi-squared test of heterogeneity was not significant from 3 studies (*P* = .51). Fixed-effect statistical model was adopted. PD in the HAIC group was less than that in the TACE group with a combined OR of 0.32 (95% CI, 0.20, 0.52; *P* < .05).

### 3.4. OS

#### 1.3.4. 1-year OS.

**Figure 3.**
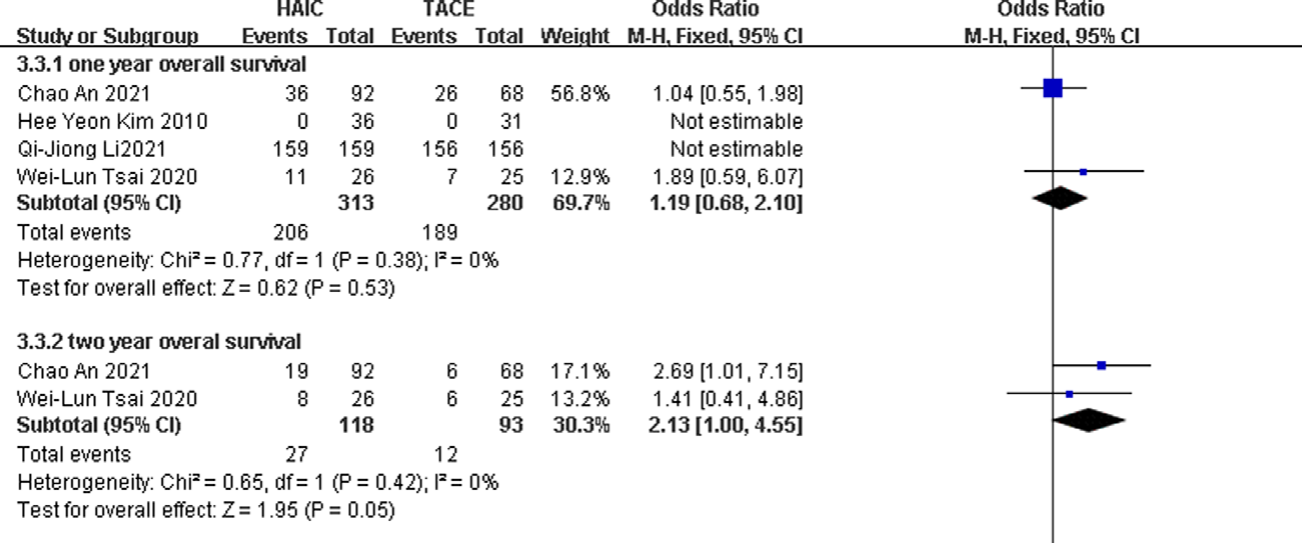
Fixed-effects statistical model of odds ratio (OR) for overall survival (1- and 2-year overall survival) after HAIC versus TACE.

The chi-squared test of heterogeneity was not significant from 4 studies (*P* = .38). Fixed-effect statistical model was adopted. There was not significant difference in 1-year OS between the 2 groups with a combined OR of 1.19 (95% CI, 0.68, 2.1; *P* = .53) (Fig.3).

#### 2.3.4. 2-year OS.

The chi-squared test of heterogeneity was not significant from 2 studies (*P* = .42). Fixed-effect statistical model was adopted. There was not significant difference in 2-year OS between the two groups with a combined OR of 2.13 (95% CI, 1.00, 4.55; *P* = .05).

#### 3.3.4. Safety.

**Figure 4.**
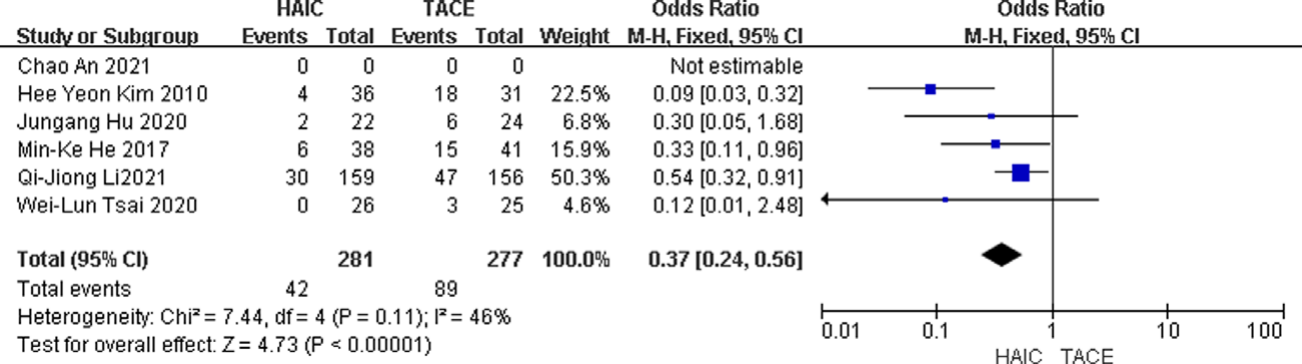
Fixed-effects statistical model of odds ratio (OR) for SAEs after HAIC versus TACE.

Common Adverse events (AEs), such as pain, vomiting, fever, nausea, happen usually in the HAIC group or TACE group and can be controlled by medical treatment, but not the same as SAEs including death, progressive deterioration of liver function or liver failure. Six studies were included. The chi-squared test of heterogeneity was not significant (*P* = .11). Fixed-effect statistical model was used. SAEs in the HAIC group was less than that in the TACE group with a combined OR of 0.37 (95% CI, 0.24, 0.56; *P* < .05) (Fig. 4).

### 3.5. Sensitivity analysis and publication bias

The data of ORR, PR, SD, DCR, PD, 1-year OS, 2-year OS or SAEs was conducted using the fixed-effect or random-effect statistical model, respectively. The results were similar and the combined results were highly reliable.

The funnel plot of Begg’s test exhibited symmetricalness. There were not publication bias in the study, which suggesting that the results of this meta-analysis are statistically reliable.

## 4. Discussion

In eastern/south-eastern Asia and in Africa, it is very high for HCC rate.^[24]^ In China, HCC is the second most common malignant tumor and about half of the new patients in the world are Chinese, and about 300,000 to 400,000 people die due to HCC every year.^[25,26]^ A lot of HCC were unresectable when initially diagnosed. Large or giant HCC is usually associated with filtering pathological features, namely microvascular or macrovascular infiltration.^[27–29]^ In imaging, it often shows that the tumor edge is not smooth and there is macrovascular invasion.^[30,31]^ The median survival time of patients with HCC and PVTT was only 2.7 months^[32]^ and that with unresectable HCC was less than 6 months if left untreated.^[33,34]^ More better treatments including local treatment and systemic treatment must be found. HAIC or TACE which was local treatment subsequently emerged. TACE has been widely used in the treatment of advanced or unresectable HCC. And it has long-term clinical effects and provides an opportunity for patients with unresectable HCC.

HAIC can directly deliver high-dose anticancer drugs to detected HCC or undetected micrometastasis. Importantly, HAIC has been reported to be effective in reducing the incidence of intrahepatic metastasis in these patients.^[35]^ A randomized phase III study showed a significant difference in the surgical conversion rate between the 2 groups (*P* < .004).^[36]^ In Asia, especially in Japan and South Korea, HAIC has been used as a method to improve the prognosis of advanced HCC and has been included in treatment guidelines.^[37]^ However, HAIC may be underestimated because of the small sample size of previous studies and the lack of larger randomized trials.

This meta-analysis has showed that HAIC group has more obvious advantages comparing with TACE group in ORR, PR, SD, DCR, PD, 1-year OS, 2-year OS, and SAEs. But, there were 2 studies which were categorized as “not estimable” in 1-year OS. Kim et al^[19]^ showed that overll survival was longer in HAIC group than that in TACE group, but median survival was only 193 days versus 119 days, which were less than 1 year, so none had events at 1 year OS and it was categorized as “Not estimable.” Li et al ^[22]^ reported the median OS of 23 vs 16.1 months, which were both more than 1 year, so all patients had events at 1 year overall survival and it was also categorized as “Not estimable.” This study cannot be stratified further according to other possible confounding factors, such as tumor size, median OS, the dose of HAIC, location of portal vein thrombus. Academic journals in English language from Medline, EMBASE and PubMed were searched only, so the sources of data were narrowed and there was a selection bias. Although this meta-analysis included only one randomized controlled trial and 5 nonrandomized controlled trials, after excluding the randomized controlled trial^[22]^ which occupied over half of total patients, the results of this meta-analysis were not influenced.

However, this meta-analysis has the following advantages: To the best of our knowledge, this is the first meta-analysis to compare HAIC with TACE. The primary outcomes are higher reliability than the published randomized or nonrandomized controlled trials.

## 5. Conclusion

The study showed that HAIC is superior to TACE in Patients with unresectable HCC. In order to evaluate the long-term efficacy of HAIC and improve its stability, it is necessary to design additional rigorous, multicenter, large sample randomized controlled trials and use reliable methodology.

## Author contributions

**Conception of the study:** Yijun Wang, Guiming Shu, Cheng Lou, and Zhi Du.

**Analysis and manuscript preparation:** Junguo Liu, Jinjuan Zhang.

**Performed the data analyses and wrote the manuscript:** Junguo Liu.

**Helped perform the analysis with constructive discussions:** Junguo Liu, Jinjuan Zhang, Yijun Wang.

**Contributed fund:** Jinjuan Zhang and Guiming Shu.
